# Supplementary figures and images for: Anti-ENO1 antibody combined with metformin against tumor resistance: a novel antibody-based platform
Source: PeerJ. 2024 Mar 18;12:e16817. doi: 10.7717/peerj.16817 (PMC10956521; doi:10.7717/peerj.16817)

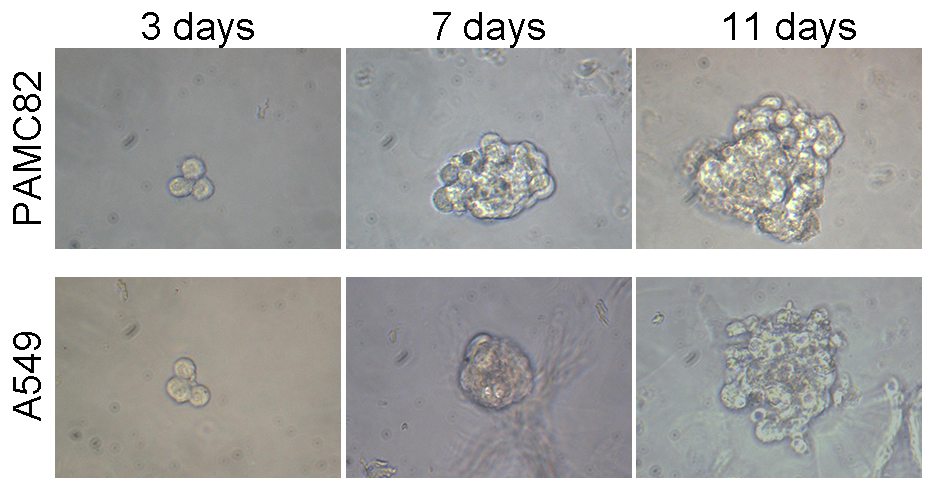

Supplement: Supplemental Information 1 [file peerj-12-16817-s001.jpg]

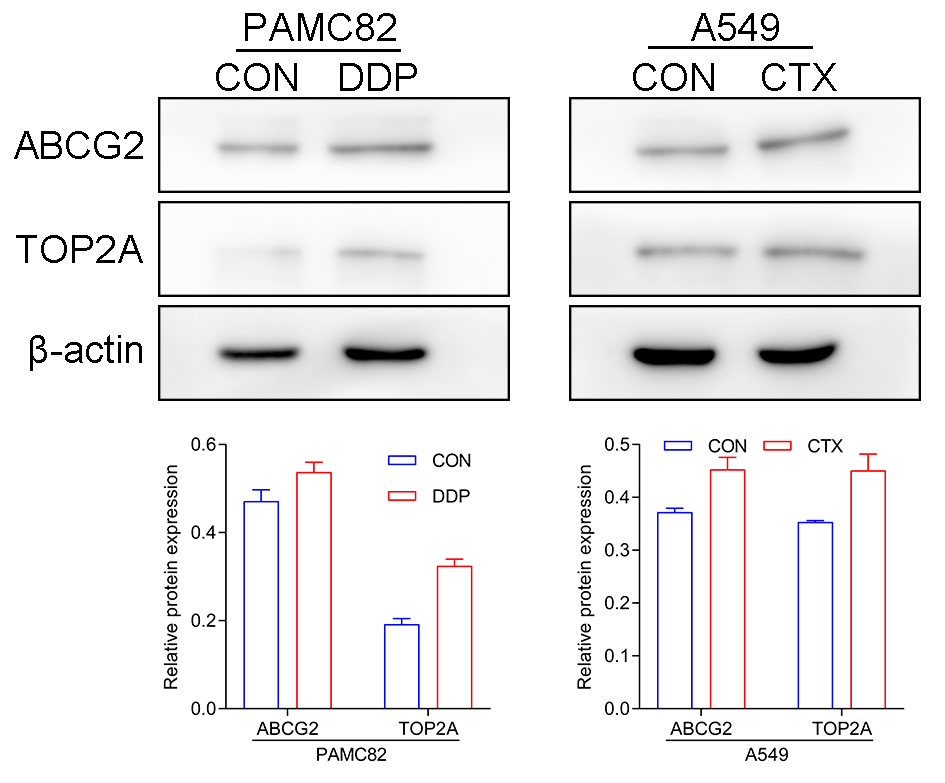

Supplement: Supplemental Information 2 [file peerj-12-16817-s002.png]

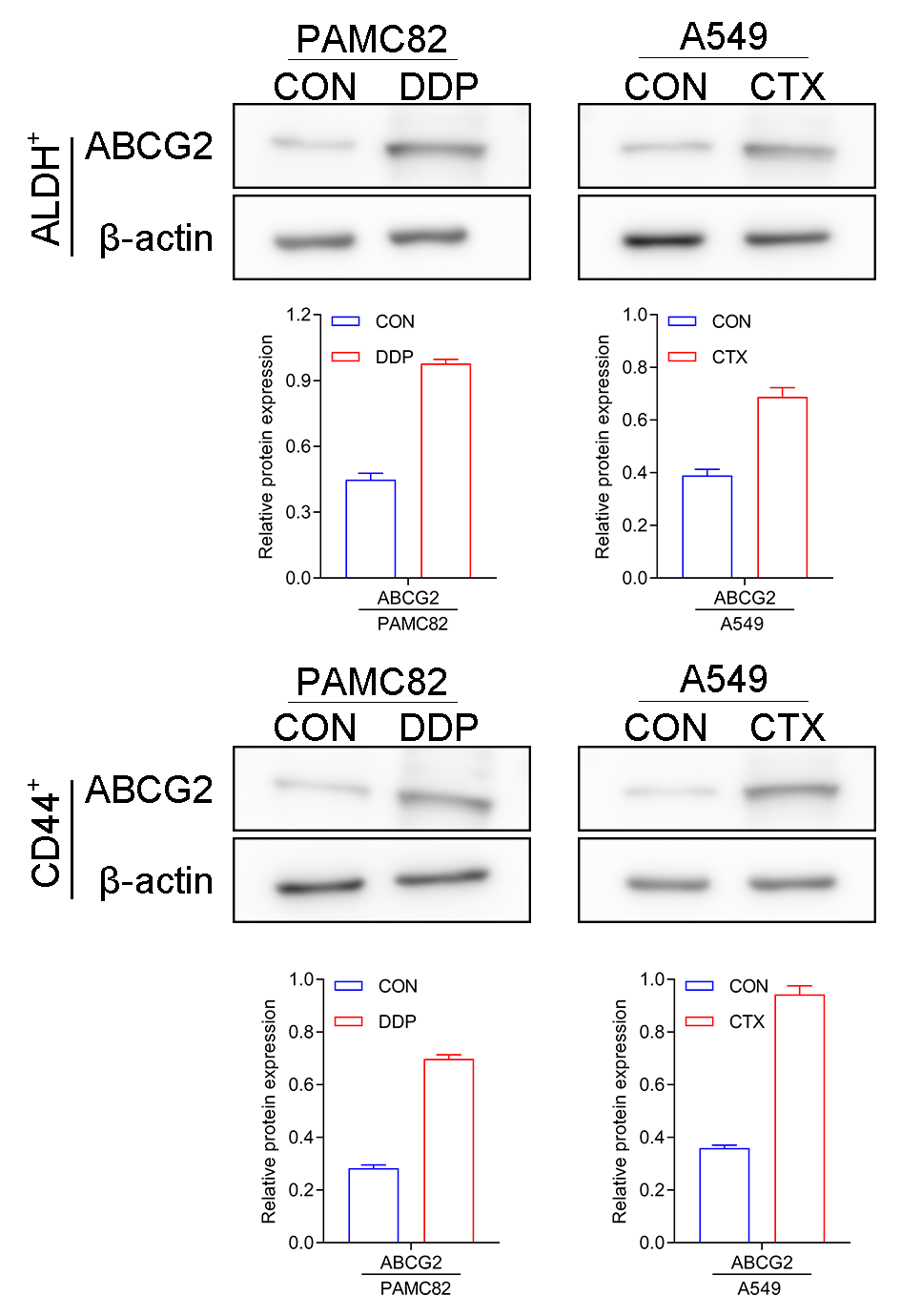

Supplement: Supplemental Information 3 [file peerj-12-16817-s003.png]

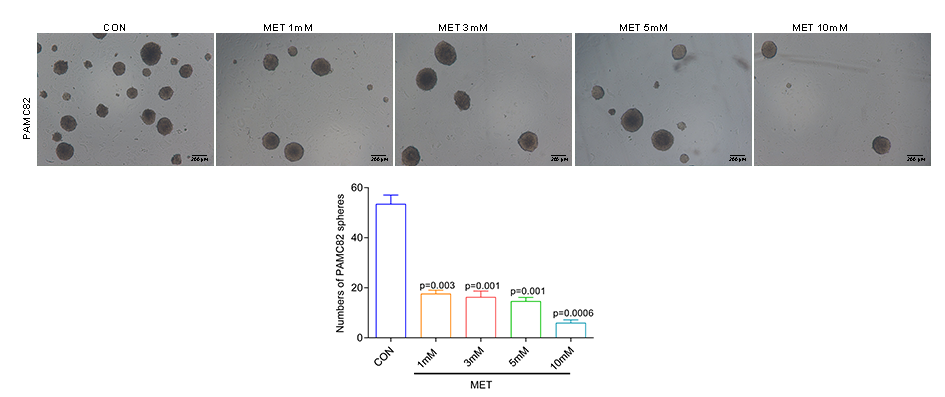

Supplement: Supplemental Information 4 [file peerj-12-16817-s004.png]

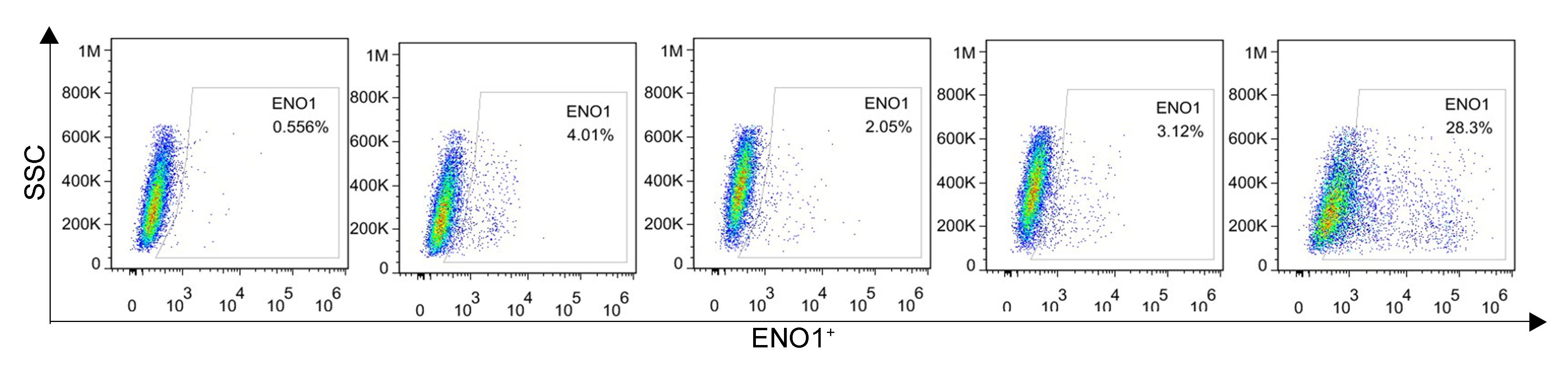

Supplement: Supplemental Information 5 [file peerj-12-16817-s005.jpg]

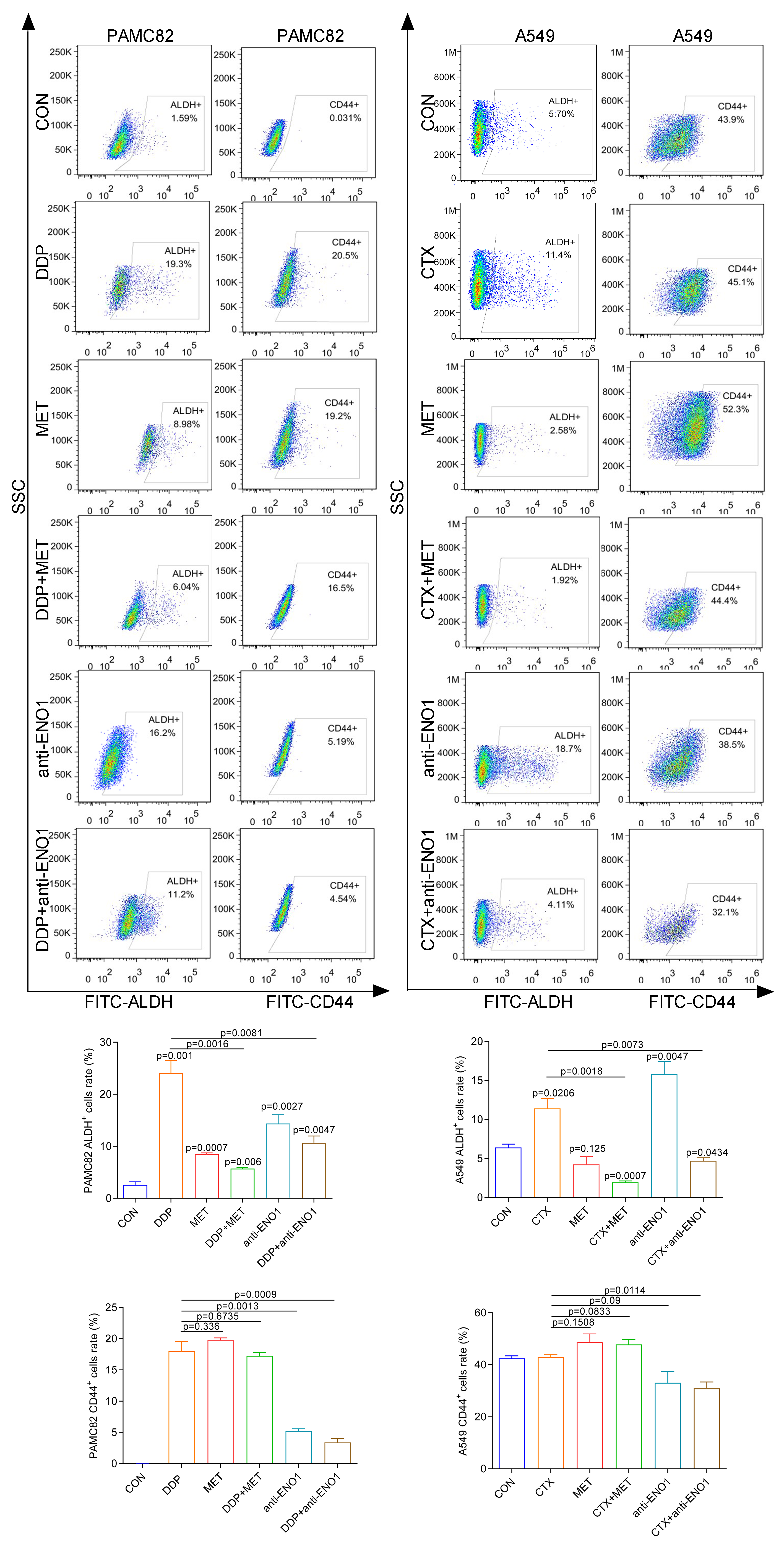

Supplement: Supplemental Information 6 [file peerj-12-16817-s006.png]

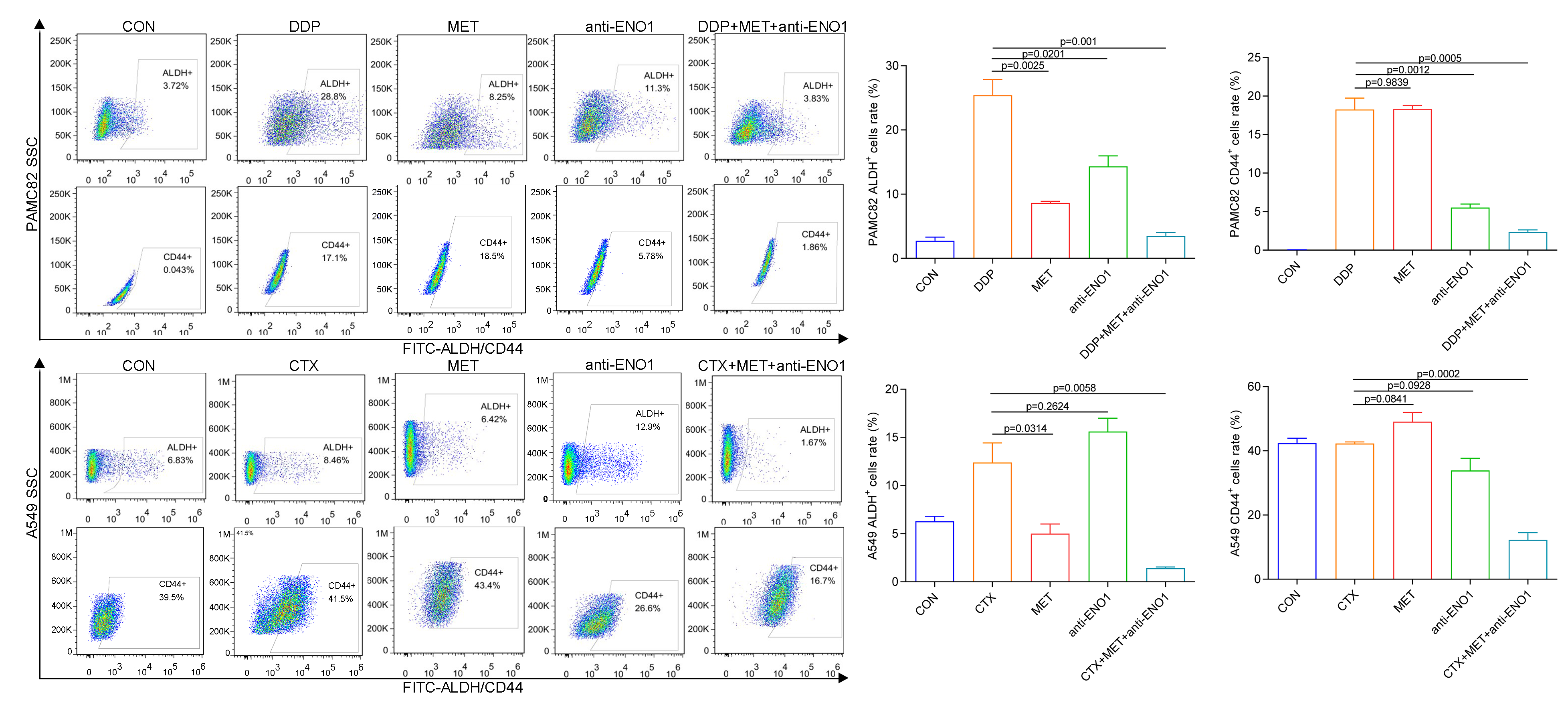

Supplement: Supplemental Information 7 [file peerj-12-16817-s007.png]
