# Supplementary material for: Anti-ENO1 antibody combined with metformin against tumor resistance: a novel antibody-based platform
Source: PeerJ. 2024 Mar 18;12:e16817. doi: 10.7717/peerj.16817 (PMC10956521; doi:10.7717/peerj.16817)
Supplement: Supplemental Information 8 [file peerj-12-16817-s008.docx]

**Supplementary file.** Complete blot images for Figure 3 and Figure 5

**Figure 3 (left):**

**PMAC82 p-AMPK PMAC82 AMPK PMAC82 β-actin**









**A549 p-AMPK A549 AMPK A549 β-actin**









**Figure 3 (right):**

**PMAC82 p-AKT PMAC82 AKT PMAC82 β-actin**









**A549 p-AKT** **A549 AKT** **A549 β-actin**









**Figure 5 (left):**

**PMAC82 AMPK** **PMAC82 p-AMPK**  **PMAC82 AKT** **PMAC82 p-AKT**











**PMAC82 GSK3β** **PMAC82 p-GSK3β PMAC82 β-catenin PMAC82 p-β-catenin**











PMAC82 Cyclin D1 PMAC82 β-actin







**Figure 5 (right):**

A549 AMPK A549 p-AMPK A549 AKT A549 p-AKT











A549 GSK3β A549 p-GSK3β A549 β-catenin A549 p-β-catenin











A549 Cyclin D1 A549 β-actin
